# Fresh fruits, vegetables and mushrooms as transmission vehicles for *Echinococcus multilocularis* in highly endemic areas of Poland: reply to concerns

**DOI:** 10.1007/s00436-016-5149-4

**Published:** 2016-06-01

**Authors:** Anna Lass, Beata Szostakowska, Przemysław Myjak, Krzysztof Korzeniewski

**Affiliations:** 1 Department of Tropical Parasitology, Institute of Maritime and Tropical Medicine in Gdynia, Medical University of Gdansk, 9b Powstania Styczniowego Str., 81-519 Gdynia, Poland; 2 Epidemiology and Tropical Medicine Department in Gdynia, Military Institute of Medicine in Warsaw, Grudzińskiego St. 4, 81-103 Gdynia, Poland

**Keywords:** *Echinococcus multilocularis*, Fruits, Vegetables, Mushrooms, Contamination

## Abstract

*Echinococcus multilocularis* is a tapeworm that may cause alveolar echinococcosis (AE), one of the most dangerous parasitic zoonoses. As in the case of other foodborne diseases, unwashed fruits and vegetables, contaminated with dispersed forms of *E. multilocularis*, may serve as an important transmission route for this parasite. In this article, we reply to the incorrect interpretation of results of our study concerning the detection of *E. multilocularis* DNA in fresh fruit, vegetable and mushroom samples collected from the highly endemic areas of the Warmia-Masuria Province, Poland, to dispel any doubts. The accusations formulated by the commentators concerning our paper are unfounded; moreover, these commentators demand information which was beyond the purview of our study. Making generalisations and drawing far-reaching conclusions from our work is also unjustified. The majority of positive samples were found in only a few hyperendemic communities; this information corresponds with the highest number of both infected foxes and AE cases in humans recorded in this area. Our findings indicate that *E. multilocularis* is present in the environment and may create a potential risk for the inhabitants. These people should simply be informed to wash fruits and vegetables before eating. No additional far-reaching conclusions should be drawn from our data. We believe these commentators needlessly misinterpreted our results and disseminated misleading information. Nevertheless, we would like to encourage any readers simply to contact us if any aspects of our study are unclear.

*Echinococcus multilocularis* is a cestode species of the genus *Echinococcus* which may cause alveolar echinococcosis (AE), one of the most dangerous parasitic zoonoses, characterised by a high fatality rate. The incubation time of the disease can vary between less than 5 and 15 years. The initial phase is always asymptomatic. The number of AE cases is increasing worldwide. In Poland, AE has shown an increasing morbidity and mortality rate since 1990. Until 2011, Poland was the fourth European country to experience more than 120 AE cases (Głuszcz and Kałczak 1960; Wesołowski et al. 1970; Kern et al. 2003; Nahorski et al. 2013). In Europe, the red fox plays a role as the main definitive host of this tapeworm (Eckert and Deplazes 2004; Karamon et al. 2014). In Poland, various studies have shown not only the presence of foxes but also a distinct and dynamic increase in both the fox population (by a factor of about four) and the percentage of infected foxes (by a factor of three) over the past 15–20 years (the same situation has been observed in other endemic regions of Europe) (Karamon et al. 2014). The highest level of *E. multilocularis* prevalence (50 %) was observed in the Warmia-Masuria Province (north-eastern Poland) where the greatest number of human AE cases has been noted (Karamon et al. 2014; Nahorski et al. 2013). People may be infected by the ingestion of *E. multilocularis* eggs excreted with the faeces of the definitive hosts. It is generally believed that humans may be exposed to tapeworm eggs via contact with infected animals or contaminated food or environments. However, the significance of the various potential routes of transmission remains unknown (Eckert and Deplazes 2004; Kern et al. 2004). To date, there is a lack of data about the occurrence of *E. multilocularis* eggs on fresh plants intended for consumption. However, data from different parts of the world, including Poland, show that fruits and vegetables may be vehicles for many parasites, which can be found in different numbers depending on the endemicity of the region and other factors (Shahnazi and Jafari-Sabet 2010; Kłapeć and Borecka 2012; Lass et al. 2012; Said 2012; Chau et al. 2014; Duedu et al. 2014).

In our last paper, we published the results of our preliminary study on the contamination of fresh fruits, vegetables and mushrooms with *E. multilocularis* DNA in chosen areas of the Warmia-Masuria Province, Poland (Lass et al. 2015). The parasite DNA was detected in 23.3 % of the samples tested; the majority of positive samples were found in a few highly endemic communities (demonstrating the highest AE rate, as well as the large number of infected foxes in Poland). Therefore, we indicated a potential source of human infections.

However, recently, some doubts have been formulated by Robertson et al. (2016). Therefore, we reply to these accusations in this paper in order to dispel any doubts and avoid further misinterpretation of our results.

Epidemiological data presented in the paper by Robertson et al. (2016) are random and incomplete, and thus, cannot serve as a background for the results of our studies (Lass et al. 2015). The epidemiological situation in Poland concerning the prevalence of *E. multilocularis* and numbers of AE cases differs significantly between particular provinces. The fullest data can be found in a paper by Nahorski et al. (2013), in which the most prominent Polish researchers and physicians describe the problem of alveococcosis in Poland, based on analysis of all (not randomly selected) autochthonous human AE cases recorded in Poland in the years 1990–2011. The authors showed that the highest number of AE cases (53.7 %) was recorded in the Warmia-Masuria Province (north-eastern Poland); accordingly, this region was taken into consideration in our study (Lass et al. 2015). The criterion of high endemicity (local annual incidence of two or more AE cases per 100,000 inhabitants) was fulfilled by four communities of the Warmia-Masuria Province (detection rate per 100,000 inhabitants: Srokowo 5.3, Barciany 2.02, Budry 3.9 and Kiwity 2.59), characterised by sparsely populated woodland, with a large population of red foxes (*Vulpes vulpes*) and raccoon dogs (*Nyctereutes procyonoides*). It should be emphasised that the average detection rate for human AE in the Warmia-Masuria Province in the years 1990–2011 was 0.20, as compared with 0.014 per 100,000 inhabitants in the entire country. This shows that the Warmia-Masuria Province cannot be evaluated based on general information concerning Poland as a whole. Importantly, Nahorski et al. (2013) state that the actual number of human AE cases in Poland is presumably much higher than officially reported. Unreported cases may include non-diagnosed (probably due to diagnostic difficulties) AE cases, misdiagnosed cases treated usually as primary or secondary malignancies of the liver, cases associated with the imprecise diagnosis of ‘echinococcus-caused disease’, and true AE cases not reported to the State Epidemiological Sanitary Inspection, despite the existing legal obligation to report all AE cases. Therefore, general reports of the State Epidemiological Sanitary Inspection and National Health Institute may be not complete. Robertson et al. (2016) suggest that the high number of environmental samples positive for *E. multilocularis* is unreliable because of, *inter alia,* the low number of AE cases, referring to information from the report of the National Health Institute (Gołąb and Czarkowski 2014) that in 2012 in Poland, seven AE cases were recorded (equivalent to 0.018/100,000) and to an investigation performed by Cisak et al. (2015), in which inhabitants of rural areas of Poland were serologically tested for *E. multilocularis*. These data do not reflect the epidemiological situation in highly endemic areas of the Warmia-Masuria Province (as described above) and provide misleading information. For example, in our clinical centre in Gdynia (Department of Tropical and Parasitic Diseases at the Medical University of Gdańsk), one of the few specialised clinical centres where most AE patients are treated in Poland, seven AE cases were diagnosed in 2012 (clinical data). Moreover, in 2012–2015, a total of 24 new AE cases were confirmed, with over 62 % (15/24) from the Warmia-Masuria Province (data not published). This highlights a problem with regular and full registration of all the detected cases of AE in Poland. In a study performed by Cisak et al. (2015), a group of 172 rural inhabitants from eastern Poland was examined in 2011. Three serum samples were positive for *E. multilocularis*; however, no detailed information concerning these patients was provided. Moreover, the majority of individuals investigated by Cisak et al. (2015) were inhabitants of the region of Lublin (136/172; 79 %) where the average number of foxes infected with *E. multilocularis* is over two times less than in the Warmia-Masuria Province (18 %) (Karamon et al. 2014), and three times less than in some highly endemic communities of the Warmia-Masuria Province; 10 individuals lived in the Białystok region, Podlaskie Province, and 26 in the Rzeszów region, Subcarpathian Province, with average levels of infected foxes of 34 and 47 %, respectively (Karamon et al. 2014). Thus, the results of a study of these particular populations should not be generalised, especially since they have nothing in common with the Warmia-Masuria rural population, particularly with inhabitants of the highly endemic areas on which our study focussed (Lass et al. 2015). Serological screening of people living there (with special attention paid to individuals who have regular, long-lasting contact with the environment) is clearly needed, but has been yet not performed.

It is also known that in Poland, the highest infection rate among red foxes (an average of 50 % of the population) is observed in the Warmia-Masuria Province (in some districts, such as the Kętrzyn district, over 62 % of foxes are infected with *E. multilocularis*) (Malczewski et al. 2008a, b; Karamon et al. 2014). Of examined foxes, 24 % showed a high intensity of infection (101–1000 tapeworms per animal), while 64 % were infected with less than 100 tapeworms (Malczewski et al. 2008a, b). These data suggest that contamination of the environment with *E. multilocularis* eggs may be locally high. The majority of Polish AE patients live in rural areas have long-term occupational and/or environmental contact with forest areas (they go bilberry-picking and mushroom-hunting), and have dogs (Nahorski et al. 2013). A high number of AE cases in a limited area, combined with a large population of infected foxes, suggests a risk to humans due to the high level of environmental contamination with tapeworm eggs and to the behaviour of the inhabitants (regular contact with the environment) (Gawor 2015). This is in agreement with the results of our study (Lass et al. 2015). We found DNA of *E. multilocularis* near the houses of AE patients (yards, kitchen gardens), not only in plant and mushroom samples but also in various environmental matrices including soil, air and water. For example, in the case of one of the patients from the Warmia-Masuria Province diagnosed in 2011, we detected *E. multilocularis* DNA in the same year in his kitchen garden/yard (in different environmental matrices), which suggest that foxes living there were infected (data not published).

A situation similar to that in the Warmia-Masuria Province has been observed in nearby Lithuania, where a total of 179 AE cases were registered between 1997 and 2013; the incidence of AE varied from 0.03 per 100,000 inhabitants in 2004 to 0.57 in 2009 and 0.74 in 2012. Of these AE patients, 81 % were farmers or people otherwise involved in agricultural activities (Marcinkutė et al. 2015). Moreover, 58.7 % of red foxes in Lithuania were found to be infected with *E. multilocularis* (Bružinskaitė-Schmidhalter et al. 2012).

A rate of 23 % positive samples of fruits, vegetables and mushrooms seems high, but is probable given the complete background. Firstly, distribution of contamination was not equal in the communities/districts investigated, as shown in our article (Lass et al. 2015). Forests and fields are natural environments for foxes; therefore, it is not surprising that *E. multilocularis* DNA was detected in such locations. Most positive samples (over 62 %) collected from the Warmia-Masuria Province were recorded in the Kętrzyn, Węgorzewo and Iława districts, including communities regarded as highly endemic, which shows that, within the endemic region of the Warmia-Masuria Province, there may be several foci characterised by certain favourable factors that have an impact on the level of contamination of the environment (and the permanent high rate of AE cases). The formation of macrofoci and microfoci characterised by intensive transmission (‘hot spots’) has been described previously, i.e. in Germany, Switzerland and France (Deplazes et al. 2004). Secondly, sampling sites were not accidental and were chosen with the help of local inhabitants (farmers, hunters, woodsmen) who showed us the locations of foxes’ lairs in forests and fields, sites favoured by foxes for defecation, and places (including yards and kitchen gardens) where foxes were most often seen (in some communities regularly or even daily). Therefore, it is not very surprising that plants collected in communities where the permanent presence of foxes had been confirmed were contaminated. Of course, detection of DNA of the parasite is evidence for its presence in the environment and indicates a potential risk for humans, but it cannot show the level of this risk. We did not calculate the infective load of positive samples (intensity). Nested PCR (used in our study) is not suitable for the performance of quantitative analysis. The microscopic method is even less suitable for this task (counting eggs), since *E. multilocularis* eggs cannot be distinguished from other taeniid tapeworm eggs that infect animals and humans and that may be present in the environment. It is also obvious that detection of DNA does not enable calculation of the viability of parasites or, consequently, their ability to infect humans. We never suggested that we had detected infective eggs, only DNA. We did not perform viability tests mainly because this was not the aim of our project and would not be covered financially. Nevertheless, we agree with Robertson et al. (2016) that such data would be valuable.

We collected material (from kitchen gardens, outdoor cultivation and forests) between June 2011 and September 2012, but not year-round as Robertson et al. (2016) suggest. It is clear that we were able to collect fresh fruits, vegetables and mushrooms only during the vegetation period of plants listed in the article (Lass et al. 2015). Indeed, a study in Zurich demonstrated a higher percentage of infected foxes in winter. However, we could not collect samples in the winter for the simple reason that no fresh fruits, vegetables or mushrooms could be found at that time in Poland in the forest or in kitchen gardens. Most samples were collected during spring, early summer and autumn.

We took all necessary steps to avoid any contamination of collected material or laboratory contamination. Each sample was transported in a separate disposable bag and was treated individually in the laboratory with all necessary precautions. Particular areas for sample proceedings were well separated: (1) a laboratory for initial procedures (i.e. egg recovery), (2) rooms for material storage, and (3) a professional laboratory for molecular studies (with separate rooms for DNA extraction, PCR mixture preparation, DNA loading, PCR processing and electrophoresis). All areas are UV-sterilised. Moreover, all PCR experiments were performed with positive and negative controls (Lass et al. 2015). Furthermore, prior to the start of our project (investigation of samples from rural areas), over 100 samples bought in big supermarkets were analysed for *E. multilocularis*; all of them were found negative. This information was not included in our paper because in our opinion, it was not necessary; however, this test showed that our results were correct. Therefore, we did everything that should have been done to avoid contamination; thus, the doubts of the commentators (Robertson et al. 2016) are unfounded.

In our paper, we showed that *E. multilocularis* DNA was found in 21 % of samples from forests, 30.7 % of samples from kitchen gardens and 20 % of samples from plantations (Lass et al. 2015). Therefore, the statement of Robertson et al. (2016) that more positive samples were found in raspberry samples than in low-growing samples (i.e. from kitchen gardens) is false. Our results are clearly the opposite. Moreover, the commentators claim that the percentage of positive samples from plantations of raspberries is high and unreliable because raspberries are elevated above the ground in comparison to other plants. The point is that we did not collect fruit from the tops of raspberry bushes but from the lowest branches, as it was clear to us that this location would be most exposed to contamination with tapeworm eggs. This sampling detail was not provided in our paper, an omission which might give rise to doubt; therefore, we are explaining this now. What is more, we also collected air samples (1000 L each) from the sampling area from a height of about 30–50 cm from the ground. *E. multilocularis* DNA was present in the air samples (especially when fox burrows and fox faeces were present in the surrounding area) (paper in preparation). This may suggest that eggs may be spread from the ground (from fox faeces) by the wind and may contaminate surrounding plants. Also importantly, these samples were not taken from huge commercial plantations but from small local plantations (cultivations) in close proximity to forests. Therefore, the suggestion of the commentators that positive results (in raspberries) could result in some economic losses in the agricultural section is superstitious, especially considering the relatively small number of samples investigated and the fact that we detected DNA only, not viable eggs.

The next set of doubts of Robertson et al. (2016) concerns our finding of positive samples of forest fruits and mushrooms in close proportion to samples from plantations (the authors suggest a low number of *E. multilocularis*-infected intermediate hosts in forests). This doubt is unfounded because in our paper (Lass et al. 2015), only general information about sampling sites was provided. In fact, we did not collect samples from the middle of the forests but from the edges, bordering on grasslands, in compliance with the aim and scope of our project. This detailed data should dispel the doubts of the commentators. It is also worth mentioning that fox faeces from grassland and forests borders were positive for *E. multilocularis*, while faeces taken from the middle of the forest were negative. On the other hand, in Poland, it is not clearly established which intermediate hosts play a role in the transmission of the parasite. In the course of an investigation of a large number of intermediate hosts, no *E. multilocularis*-infected individuals were found (Malczewski et al. 2008a, b). Therefore, this aspect requires further study.

The same methodology described in our article (Lass et al. 2015) was applied to check environmental contamination in the non-endemic Pomerania Province, which neighbours the Warmia-Masuria Province. Using the same methods and the same ‘sampling key’, we found plants and mushroom samples to be over 3.5 times less contaminated than in the highly endemic districts of the Warmia-Masuria Province (manuscript in preparation). This confirms the correctness of our methods. However, improvement of the methodology for detection of *E. multilocularis* is desirable.

We also disagree with the suggestion that, since we detected high numbers of positive plant samples in some localities, the extent of infection with *E. multilocularis* in the Polish population in this area should be much greater than indicated by the published data. Indeed, the number of cases reported from hyperendemic areas of the Warmia-Masuria Province (where the majority of positive samples were collected) is very high (AE rate 2 to 5/100,000 inhabitants). Fifteen new AE cases from the Warmia-Masuria Province within the last 3 years (cases from our clinic in Gdynia) are disturbing enough. Moreover, there is no serological screening data from these communities; thus, the actual condition of this population is unknown. It is also highly likely that the number of eggs present on plants may change (for example, eggs might be washed by rain from the surface of plants) and other measurements in the same area could produce different results. Therefore, the percentage of contaminated plants (Lass et al. 2015) we presented cannot be regarded as stable. We plan a long-lasting study focused on hyperendemic areas in the future, with successive monitoring of sampling sites. Without this data, we do not presume to speculate on how severe or frequent the threat is for humans. However, it should be taken into consideration that in Poland, distinct and dynamic increases in the fox population (by a factor of about four) and in the percentage of infected foxes (by a factor of about three) over the past 15–20 years have been observed (Karamon et al. 2014), which clearly must influence the level of contamination of the environment. Moreover, the number of infections may correlate not only with the level of environmental contamination but also with the inhabitants’ behaviour (as it was shown that long-term, often occupational contact with the environment plays a role). It is also known that humans have a relatively high degree of innate resistance to infection by the eggs of *E. multilocularis*. Pathological features and the frequent absence of protoscoleces in AE lesions suggest that humans are generally ‘poor hosts’ for *E. multilocularis* (Vuitton et al. 2006). Fortunately, the number of established AE infections in humans is far smaller than the number of humans exposed to the parasite eggs. It is estimated that only one out of ten subjects exposed to oncospheres of *E. multilocularis* will be subject to development of the metacestode and will thus develop AE lesions (Gottstein et al. 2001). The incubation time of the disease varies from less than 5 to 15 years, but the shortest period between oral uptake of *E. multilocularis* eggs and seroconversion is unknown. Moreover, not all humans react similarly when infected with *E. multilocularis*. Clustering of cases in certain families and in communities exposed to similar risk factors also points to genetic predisposition factors (Vuitton et al. 2003; Yang et al. 2005; Yang 2005).

In summary, we have found *E. multilocularis* DNA in plant and mushroom samples collected from the territory of the endemic Warmia-Masuria Province, Poland (Lass et al. 2015) and have shown that the majority of positive samples were found in a few highly endemic localities. Considering the large number of infected foxes in this area, the large areas of grassland (which favours the population of intermediate hosts susceptible to *E. multilocularis*), the highest number of AE cases noted there (in the past and present) and the specific ‘sampling key’ in compliance with the main aim of our work (sampling sites at the highest potential risk of contamination with eggs), the results of our study are not surprising. It needs to be highlighted that the number of samples investigated was insufficient for drawing any serious conclusions except one—which we stated—namely, that the DNA of *E. multilocularis* found in fruit, vegetable and mushroom samples indicates the presence of this parasite, and therefore. eating unwashed items may create potential risk for humans. That is all. The risk level remains unknown, but lack of knowledge about the number of eggs in a sample or their viability (however useful) does not obviate the risk to humans. Therefore, people living in highly endemic areas should be aware of the potential threat; they need not avoid eating fresh fruits and vegetables, but should simply wash them prior to consumption. Our study was preliminary; the results suggest that more studies should be performed in future (especially focussed on highly endemic/highly contaminated communities) including a larger number of tested samples and long-term monitoring of the environment to obtain a clearer picture of reality. Drawing far-reaching conclusions based on the results of our preliminary study is a form of misinterpretation.

In our opinion, the accusations formulated by the commentators on our work are unfounded and their conclusions excessive. Disregard for the potential risk for humans living in highly endemic foci (especially those maintaining regular long-term contact with the environment) may bring fatal consequences. Treatment of AE is not refunded in Poland, and unfortunately infected people are often very poor (communities in the Warmia-Masuria Province are currently experiencing the highest unemployment rate in the country) and cannot afford the cost of the long treatment. Nahorski et al. (2013) demonstrate the need to increase public awareness of the potential for contracting *E. multilocularis* and of the disease’s consequences, and the necessity for training primary care physicians in recognising the risk of AE in order to enable early detection of this dangerous disease. This is consistent with the recommendations developed at the Swiss International Exploratory Workshop ‘Alveolar Echinococcosis in Poland, Lithuania and Switzerland’, Zurich, 17–19 November 2010 (unpublished report). We actively participate in these activities and will redouble our efforts to prepare an effective prophylactic educational programme addressed to the people from highly endemic communities who are the most exposed to the threat. Unfounded questioning of our work can make this more difficult.

We would like to encourage our readers to contact us if any aspects of our study/article are unclear or insufficiently highlighted before making public accusations.
